# Mortalidade em Doenças Cardíacas Congênitas no Brasil - o que sabemos?

**DOI:** 10.36660/abc.20200589

**Published:** 2020-12-01

**Authors:** Andressa Mussi Soares

**Affiliations:** 1 Hospital Evangélico de Cachoeiro de Itapemirim Cachoeiro de ItapemirimES Brasil Hospital Evangélico de Cachoeiro de Itapemirim - Cardiologia Congênita, Pediátrica e Fetal, Cachoeiro de Itapemirim, ES - Brasil; 2 CORImagem Cachoeiro de ItapemirimES Brasil CORImagem - Cardiologia Congênita, Pediátrica e Fetal, Cachoeiro de Itapemirim, ES – Brasil

**Keywords:** Cardiopatias Congênitas, Mortalidade, Teste de Oximetria, Subdiagnóstico, Epidemiologia, Administração em Saúde Pública

A doença cardíaca congênita (DCC) compreende qualquer alteração na anatomia do coração e de seus vasos sanguíneos. A incidência de CHD é de 8 a 10 por 1.000 nascidos vivos, ou seja, 1 caso em 100 nascimentos. No Brasil nascem 28.900 crianças com DCC por ano (1% do total de nascimentos), das quais cerca de 80% (23.800) necessitam de cirurgia cardíaca, e metade delas, no primeiro ano de vida.^1^As malformações congênitas representam a segunda principal causa de mortalidade em crianças menores de um ano. A DCC é a mais frequente delas e com a mais alta mortalidade no primeiro ano de vida no Brasil, sendo a segunda causa de morte até 30 dias de vida.^2^ As manifestações da doença cardíaca congênita são muito variáveis podendo ocorrer logo após o nascimento, ou mais tarde na infância ou adolescência.

O Brasil é um país continental, onde a diversidade tanto no diagnóstico quanto no tratamento das cardiopatias congênitas é muito grande entre as macrorregiões. É fato que houve uma melhora gradativa no diagnóstico após a disseminação mais ativa do teste de oximetria, com os avanços tecnológicos e a difusão da ecocardiografia por todo o país, mesmo assim uma baixa taxa de sobrevida ainda é observada no período neonatal. Lopes et al. demonstraram uma letalidade de 64,7% para cardiopatias congênitas críticas e uma redução da taxa de sobrevida em 28 dias de quase 70% nesses recém-nascidos, sinalizando grande necessidade de investimento em tecnologia assistiva e profissionais capacitados para essa população.^3^

Existem alguns estudos nacionais avaliando a tendência da mortalidade por malformações cardíacas no Brasil. Braga et al.,^4^ realizaram um estudo ecológico observacional com base no Sistema de Informações sobre Mortalidade (SIM), cujos dados são gerenciados pelo Ministério da Saúde, e são processados pelo Sistema de Informações do Sistema Único de Saúde (DATASUS). Os autores encontraram uma tendência de redução dos casos de mortalidade por cardiopatias congênitas no Brasil durante o estudo, mas apontam como limitação a provável subnotificação e subdiagnóstico, principalmente no período neonatal .^4^

Recentemente, no artigo intitulado “Desigualdades nas taxas de mortalidade por malformações do aparelho circulatório em menores de 20 anos nas macrorregiões brasileiras”,^5^ cujos dados do estudo foram extraídos do DATASUS e do Instituto Brasileiro de Geografia (IBGE) e ficaram diretamente relacionados à precisão do preenchimento dos certificados de óbitos. A codificação da causa básica do óbito foi realizada de acordo com a 100ª revisão da Classificação Estatística de Doenças e Problemas Relacionados à Saúde da Organização Mundial da Saúde (CID 10). Houve 1.367.355 mortes por todas as causas em crianças menores de 20 anos, sendo 55% em crianças menores de um ano. Os óbitos por malformações congênitas totalizaram 144.057, dos quais 39% foram por malformações do aparelho circulatório, correspondendo a 5,3/100 mil habitantes. Entre 2000 e 2015, a principal causa de morte em menores de 20 anos foi a malformação do aparelho circulatório.

Neste estudo, as regiões Sul e Centro-Oeste tiveram quase o dobro do risco de morte por malformação cardíaca congênita do que as regiões Norte e Nordeste em menores de um ano, com redução progressiva desse risco com o aumento da faixa etária. Esses dados reforçam mais uma vez a provável escassez diagnóstica, refletindo diretamente na notificação de dados nas declarações de óbito.^5^ Também foram destacados os elevados percentuais de diagnósticos imprecisos de malformação do aparelho circulatório, que foram classificados como não especificados nas declarações de óbito, principalmente nas regiões mais pobres do país.

Crianças que nasceram com malformações cardíacas congênitas e puderam ser tratadas em tempo hábil, podem desenvolver complicações evolutivas, como insuficiência cardíaca, endocardite infecciosa, disfunção de próteses e tubos, tromboses, arritmias cardíacas, disfunção ventricular, além da necessidade de reoperações ou novos procedimentos intervencionistas. Estas comorbidades e as reintervenções podem evoluir com um desfecho desfavorável ainda na infância, adolescência ou no início da idade adulta, aumentando ainda mais a taxa de mortalidade por doenças do aparelho circulatório em pacientes com cardiopatias congênitas.

A mortalidade proporcional corresponde à razão entre os óbitos por causas específicas e o total de óbitos. Segundo dados da *Global Burden of Disease* , a mortalidade proporcional por malformação cardíaca congênita em menores de 20 anos, em 2015, foi de 6,5% no Brasil, 9,7% no México, 5,8% na América Andina (Bolívia, Equador e Peru), 7,8% na Argentina, Chile e Uruguai e 4,4% no Caribe.^6^

Nas faixas etárias neonatal e infantil ainda ocorre um notável índice de diagnósticos imprecisos como causa de morte em todo o Brasil, sobretudo nas regiões Norte e Nordeste. Esse fato reforça que é fundamental fortalecer as estratégias de saúde pública voltadas para o diagnóstico e tratamento precoce das cardiopatias congênitas. Algumas medidas já foram tomadas, como o “pacto pela redução da mortalidade materna e neonatal” firmado entre os três níveis de atenção da Federação Brasileira em 2004; a instituição da oximetria obrigatória em 2014,^7 , 8^ que apesar de sua extrema importância e fácil execução, infelizmente ainda não é uma realidade nacional em todas as maternidades do país até o momento.^9^

Em 2017, o Ministério da Saúde brasileiro lançou um projeto federal para expandir o atendimento à criança com cardiopatia congênita,^2^ com a meta de aumentar em 30% o atendimento à criança cardiopata por ano, o que corresponde a mais de 3.400 procedimentos por ano, totalizando cerca de 12.600 procedimentos / ano, o que teria um grande impacto na redução da mortalidade neonatal. Muito precisa ser feito para minimizar a mortalidade neonatal extremamente elevada no Brasil por cardiopatias congênitas, desde a otimização do diagnóstico precoce no recém-nascido ou fetal no pré-natal, até a estruturação de leitos em unidade de terapia intensiva para tratamento desses recém-nascidos, seja por intervenção clínica, cirúrgica ou percutânea na hemodinâmica..

A associação de forças em todas as esferas precisa ganhar volume. A disseminação desse grave problema de saúde pública deve ocorrer de forma ampla para a sociedade e o almejado apoio do sistema empresarial privado nessa luta precisa ser fortalecido, como é o caso dos países mais desenvolvidos. Todos os profissionais de saúde envolvidos, médicos, enfermeiras, psicólogos, fisioterapeutas, e nutricionistas precisam estabelecer cada vez mais uma parceria ativa com o sistema público de saúde, a fim de que mais hospitais possam ser treinados em todas as etapas necessárias ao atendimento de pacientes com cardiopatias congênitas. A partir de equipes mais treinadas, melhores dados de notificação ocorrerão neste extenso Brasil, fornecendo um retrato mais confiável da realidade das cardiopatias congênitas em nosso país. Além disso, mais diagnósticos com consequentes tratamentos precoces farão toda a diferença na quebra desse grande paradigma que é a mortalidade infantil por cardiopatias congênitas em nosso país.
