# Supplementary material for: Mortalidade em Doenças Cardíacas Congênitas no Brasil - o que sabemos?
Source: Arq Bras Cardiol. 2020 Dec 1;115(6):1174–5. [Article in Portuguese] doi: 10.36660/abc.20200589 (PMC8133721; doi:10.36660/abc.20200589)
Supplement: Translation

# Translation: Mortality in Congenital Heart Disease in Brazil - What do we Know?

**Authors:** Andressa Mussi Soares

**Affiliations:** 1 Brazil Hospital Evangélico de Cachoeiro de Itapemirim - Cardiologia Congênita, Pediátrica e Fetal, Cachoeiro de Itapemirim, ES - Brazil; 2 Brazil CORImagem - Cardiologia Congênita, Pediátrica e Fetal, Cachoeiro de Itapemirim, ES – Brazil

**Keywords:** Congenital Heart Disease, Mortality, Oximetry Test, Underdiagnosis, Epidemiology, Public Health Administration

Congenital Heart Disease (CHD) comprises any change in the anatomy of the heart and its blood vessels. The incidence of CHD is 8 to 10 per 1,000 live births, that is or one in one hundred births. In Brazil, 28,900 children are born with CHD per year (1% of the total births), of which about 80% (23,800) require cardiac surgery, and half of them, within the first year of life.^1^Congenital malformations represent the second main cause of mortality in children under one year of age. CHD is the most frequent and the one with the highest mortality in the first year of life in Brazil, and the second cause of death in up to 30 days of life.^2^Congenital heart disease manifestations are very variable and may occur soon after birth or appear later in childhood or adolescence.

Brazil is a continental country, where the diversity observed both in the diagnosis and in treatment of congenital heart diseases is very large among the macro-regions. It is a fact that there has been a gradual improvement in diagnosis, after the more active dissemination of the oximetry test, with technological advances and the diffusion of echocardiography throughout the country, even so a low survival rate is still observed in the neonatal period. Lopes et al. demonstrated a lethality of 64.7% for critical congenital heart disease and a 28-day survival rate reduced by almost 70% in these newborns, signaling a great need for investment in assistive technology and trained professionals for this among this population.^3^

There have been some national studies evaluating the trend of mortality from cardiac malformations in Brazil. Braga et al carried out an observational ecological study based on the Mortality Information System (SIM), of which data are managed by the Ministry of Health and are processed by the Information System of the Unified Health System (DATASUS). The authors found a tendency toward a reduction in cases of mortality from congenital heart disease in Brazil during the study, but point out the probable underreporting and underdiagnosis as study limitations, especially in the neonatal period.^4^

Recently in the article entitled “Inequalities in mortality rates due to malformations of the circulatory system in children under 20 years of age among Brazilian macro-regions”,^5^the study data were extracted from DATASUS and the Brazilian Institute of Geography (IBGE) and were directly related to the accuracy of the filling-in of death certificates. The codification of the basic cause of death was performed according to the 100^th^revision of the World Health Organization’s Statistical Classification of Diseases and Related Health Problems (ICD 10). There were 1,367,355 deaths from all causes among children under 20 years old, 55% in children under 1 year old. Deaths due to congenital malformations were 144,057, of which 39% were due to malformations of the circulatory system, corresponding to 5.3 / 100,000 inhabitants. Between 2000 and 2015, the main cause of death in children under 20 was circulatory system malformation.

In this study, the South and Midwest regions had almost twice the risk of death from congenital heart malformation than the North and Northeast regions in those under one year of age, with progressive reduction of this risk with an increase in the age group. These data reinforces once again the probable diagnostic scarcity, directly reflecting data notification in death certificates.^5^In this study, the high percentages of inaccurate diagnoses of circulatory system malformations, which were classified as not specified in death certificates, drew attention, mainly in the poorest regions of the country.

Children who were born with congenital heart malformations and could be treated in a timely manner, may develop evolutionary complications, such as heart failure, infectious endocarditis, prosthesis and tube dysfunction, thrombosis, cardiac arrhythmias, ventricular dysfunction, in addition to the need for reoperations or new interventional procedures. These comorbidities and reinterventions can evolve with an unfavorable outcome even in childhood, adolescence or early adulthood, further increasing the rate of mortality from diseases of the circulatory system in patients with congenital heart disease.

Proportional mortality corresponds to the ratio between deaths from specific causes and total deaths. According to data from the Global Burden of Disease, the proportional mortality from congenital heart malformation in children under 20 in 2015, was 6.5% in Brazil, 9.7% in Mexico, 5.8% in Andean America (Bolivia, Ecuador and Peru), 7.8% in Argentina, Chile and Uruguay and 4.4% in the Caribbean.^6^

The neonatal and infant age group up to one year still has a noticeable high rate of inaccurate diagnoses as a cause of death throughout Brazil, but mainly in the North and Northeast regions. This fact reinforces that it is crucial to strengthen public health strategies focused on the diagnosis and early treatment of congenital heart diseases. Some steps have been taken, such as the “pact for the reduction of maternal and neonatal mortality” signed between the three levels of attention of the Brazilian federation in 2004; the institution of mandatory oximetry testing in 2014,^7 , 8^which despite its extreme importance and easy performance, unfortunately is still not a national reality in all maternity hospitals in the country to date.^9^

In 2017, the Brazilian Ministry of Health launched a federal project to expand the care of children with congenital heart disease,^2^aiming to increase the care of these children by 30% per year, which corresponds to more than 3,400 procedures per year, totaling about 12,600 procedures / year, which would have a great impact on the reduction of neonatal mortality. Much needs to be done to minimize the extremely high neonatal mortality in Brazil related to congenital heart disease, from optimizing the early diagnosis of newborns or even prenatal diagnosis to structuring beds in the intensive care unit to treat these newborns, whether through clinical, surgical or hemodynamic intervention.

The association of efforts in all spheres needs to gain volume. The spread of this serious public health problem has to occur broadly for society and the desired support of the private business system in this struggle needs to be strengthened, as is the case in more developed countries. All health professionals involved, physicians, nurses, psychologists, physiotherapists, and nutritionists need to increasingly establish an active partnership with the public health system, so that more hospitals can be trained in all the steps necessary to care for patients with congenital heart disease. Starting with more trained teams, better data notification will occur in such a large country as Brazil, providing us with a more reliable picture of the reality of congenital heart disease in our country. In addition, more diagnoses with consequent early treatments will make all the difference in breaking this great paradigm that is infant mortality from congenital heart disease in our country.
